# Proteomic Investigation of the Response of *Enterococcus faecalis* V583 when Cultivated in Urine

**DOI:** 10.1371/journal.pone.0126694

**Published:** 2015-04-27

**Authors:** Magnus Øverlie Arntzen, Ingrid Lea Karlskås, Morten Skaugen, Vincent G. H. Eijsink, Geir Mathiesen

**Affiliations:** Department of Chemistry, Biotechnology and Food Science, Norwegian University of Life Sciences, N-1432 Ås, Norway; University of Kansas, UNITED STATES

## Abstract

*Enterococcus faecalis* is a robust bacterium, which is able to survive in and adapt to hostile environments such as the urinary tract and bladder. In this label-free quantitative proteomic study based on MaxQuant LFQ algorithms, we identified 127 proteins present in the secretome of the clinical vancomycin-resistant isolate *E*. *faecalis* V583 and we compared proteins secreted in the initial phase of cultivation in urine with the secretome during cultivation in standard laboratory medium, 2xYT. Of the 54 identified proteins predicted to be secreted, six were exclusively found after cultivation in urine including the virulence factor EfaA (“endocarditis specific antigen”) and its homologue EF0577 (“adhesion lipoprotein”). These two proteins are both involved in manganese transport, known to be an important determinant of colonization and infection, and may additionally function as adhesins. Other detected urine-specific proteins are involved in peptide transport (EF0063 and EF3106) and protease inhibition (EF3054). In addition, we found an uncharacterized protein (EF0764), which had not previously been linked to the adaptation of V583 to a urine environment, and which is unique to *E*. *faecalis*. Proteins found in both environments included a histone-like protein, EF1550, that was up-regulated during cultivation in urine and that has a homologue in streptococci (HlpA) known to be involved in bacterial adhesion to host cells. Up-regulated secreted proteins included autolysins. These results from secretome analyses are largely compatible with previously published data from transcriptomics studies. All in all, the present data indicate that transport, in particular metal transport, adhesion, cell wall remodelling and the unknown function carried out by the unique EF0764 are important for enterococcal adaptation to the urine environment. These results provide a basis for a more targeted exploration of novel proteins involved in the adaptability and pathogenicity of *E*. *faecalis*.

## Introduction

Enterococci are gram-positive bacteria normally found in food, plants, soil and water, as well as in the gastro-intestinal tracts of animals and humans. While enterococci generally are non-virulent, several species are known as antibiotic resistant opportunistic pathogenic bacteria, with *Enterococcus faecalis* and *Enterococcus faecium* being the two most important clinical species. *E*. *faecalis* is one of the leading causes of hospital-acquired bloodstream and urinary tract infections (UTIs) [1, 2] and may also cause other problems such as abdominal- and wound infections and endocarditis [3]. UTIs are thought to depend on several virulence traits, including biofilm formation, the ability to adhere to host surfaces, the presence of adhesion-promoting cell-wall/cell-membrane components such as glycolipids, and mechanisms for evading the host immune system [4–8]. Interestingly, recent results indicate that *E*. *faecalis* can not only adhere to epithelial cells in the urinary tract [4], but also invade cells, leading to formation of intracellular bacterial communities in the bladder [5]. The combination of these multiple virulence mechanisms, the ability to survive in harsh conditions, and both intrinsic and acquired resistance to many antibiotics explains the high frequency of infection by *E*. *faecalis*. *E*. *faecalis* strains of different origins, including probiotic, pathogenic and laboratory strains, show the same capacity to grow in urine [6], indicating that variation in the ability to exploit urine nutrients is not a pathogenicity-determining factor.

*E*. *faecalis* V583 (hereafter referred to as V583) was the first vancomycin-resistant clinical isolate reported in the USA and the first sequenced *E*. *faecalis* strain [7, 8]. More than 25% of its genome contains mobile genetic elements, including a large pathogenicity island. V583 has been intensively studied due to its pathogenic significance [7, 8]; however, still, little is known about the proteins and molecular mechanisms that endow this bacterium with the ability to survive and grow within an infected patient. Enterococcal responses in *in vivo* infection models and under infection-relevant growth conditions such as the presence of urine, blood, bile, or antibiotics have been studied quite extensively using transcriptomics [1, 6, 9–13]. As to cultivation in urine, four studies have been published. Vebø et al. [6] studied the response of enterococcal strains with varying virulence to growth in urine and found that transcriptional responses were quite similar. Some differential regulation was observed for genes related to stress responses, energy metabolism, acquisition of trace metals, and cell envelope modification, but it was not possible to detect pathogen-specific *E*. *faecalis* genes. Hanin et al. [12] combined quantitative reverse transcription PCR with recombination-based in vivo expression technology (R-IVET) screening to study the *E*. *faecalis* V19 strain (a V583 derivative) and found 18 genes induced after growth in urine, the majority of which were (putatively) involved in transport and protein-protein interactions, or had an unknown function. Furthermore, using real-time quantitative PCR, Shepard and Gilmore [1] studied the response of targeted genes suspected to play a role in pathogenesis of *E*. *faecalis* strain MMH594. Studies with urine revealed several genes whose expression was induced with the *E*. *faecalis* endocarditis antigen (*efaA*) demonstrating the largest change in expression. Finally, Carlos et al. [14] used reverse transcription PCR to study the response of selected virulence genes in enterococcal strains of various origins and found that growth in urine resulted in strain-specific regulation of these genes.

The proteomic approach has the advantage that it includes information from the regulatory steps between transcription and the final protein product, including translation, protein translocation and protein turnover. Furthermore, proteins from different cellular locations (intracellular, extracellular, membrane) can be identified and quantified separately, providing an extra dimension to the analysis. Extracellular proteins (the secretome) are of particular interest because they are likely to play a major role in most aspects of the host-pathogen interaction. The secretome of *E*. *faecalis* under standard conditions has been analysed by Benachour et al. in 2009 [15] and Bøhle et al. in 2011 [16], leading to the identification of 38 and 69 secreted and surface-located proteins, respectively. A comparative study of the secretomes of a food and a clinical strain of *E*. *faecalis* grown on laboratory medium led to the identification of 16 and 33 extracellular proteins, respectively [17]. The membrane proteome of *E*. *faecalis* has also been mapped, identifying 102 proteins [18]. Stress responses have so far only been studied looking at the intracellular proteome, including responses to starvation [19], the presence of bovine bile [20], vancomycin treatment [21, 22], and other stress conditions such as heat, H_2_O_2_, salt, and pH [23]. Responses of the intracellular proteome to different growth conditions (aerobic, anaerobic) and exposure to mouse intestinal cells have also been studied [24].

Here, we have used a proteomics approach to study the immediate response of *E*. *faecalis* to urine, with focus on the secretome. In an attempt to identify proteins possibly involved in UTIs, we have compared proteins in culture supernatants of the clinical isolate *E*. *faecalis* V583 when cultivated in urine or in a rich laboratory medium, 2xYT. We used recently developed label-free methods for protein quantification implemented in the MaxQuant software tool [25, 26]. Using this approach allowed identification of proteins that are clearly overproduced during incubation in urine. Of the 54 detected proteins that are predicted to be secreted, 27 were found in urine, but only six of these showed quantitative differences that warrant them being qualified as urine-specific. The present approach provides a basis for more targeted exploration of proteins that govern the behaviour of *E*. *faecalis*.

## Material and Methods

### Culture conditions and harvesting for proteomics analysis

The strain used in this study, *Enterococcus faecalis* V583 (ATCC 700802) [8] was grown overnight in 2xYT medium [1% (w/v) yeast extract, 1.6% (w/v) tryptone and 1% (w/v) NaCl] at 37°C without agitation. Growth conditions used for proteome sampling were similar to those used by Vebø et al. [6] in a previously published transcriptome study. The overnight cultures were diluted in 250 ml pre-warmed 2xYT medium to an optical density (OD_600_) of ~0.01 and incubated further at 37°C without agitation until the OD_600_ reached 0.1. The culture was then divided into two and cells were collected by centrifugation (10.000 x *g*, 3 min, 37°C). The cell pellets were re-suspended to an OD_600_ of 0.02 in pre-warmed 2xYT medium or pre-warmed sterile urine, followed by incubation at 37°C, without agitation. For incubation in urine, fresh human urine was collected from four healthy individuals, pooled and sterilized as described by Vebø et al [6]. The experiment was performed in triplicate, on three different days. Fresh urine was collected on each of these days from the same donors, and similarly, fresh 2xYT was prepared every day. This approach was chosen to account for batch-to-batch variation. Samples of each culture were collected 1.5 hours after the re-suspension in 2xYT or urine. Prior to harvesting, phenylmethylsulfonyl fluoride (PMSF) to 0.1 mM final concentration was added to the cultures to prevent proteolysis during sample preparation. The bacteria were harvested by centrifugation (6.500 x *g*, 10 min, 4°C) and the supernatant was sterilized by filtration (0.2 μm) and stored at -20°C, until use.

### Preparation of secreted proteins

To collect proteins from the supernatants, 1.6 mL of the culture supernatant was thawed at room temperature and the pH was adjusted to a pH between 7 and 8 by adding 2 μl 6 M NaOH. Proteins were then precipitated using trichloroacetic acid (TCA). First, Na-deoxycholate was added to the samples to a final concentration of 0.2 mg/mL followed by 30 minutes incubation on ice. Protein precipitation was induced by addition of TCA to a final concentration of 16% (v/v), followed by incubation on ice for 20 minutes and subsequent overnight incubation at -20 °C. Precipitated proteins were collected by centrifugation (16.000 x *g*, 30 min, 4°C) and the pellets were washed twice with ice-cold acetone, before air-drying the pellets at 60°C for ~5 minutes.

### SDS-PAGE and in-gel trypsination

The dried protein pellets were dissolved directly in NuPAGE LDS sample buffer (Invitrogen) before SDS-PAGE. Sample loading was normalized so that the amount of protein loaded corresponded to approximately the same amount of colony forming units (approximately 2.8x10^8^ CFU) for all lanes. Proteins were separated by SDS-PAGE using a 12% NuPAGE Novex Bis-Tris gel and MOPS running buffer (Invitrogen), and the gel was stained using Coomassie Brilliant Blue R250 (Bio-Rad Laboratories, Hercules, CA). Each gel lane was cut into 10 pieces and proteins therein were reduced by incubating the gel pieces in 10 mM DTT at 56°C for 30 minutes and further alkylated using 55 mM iodoacetamide at room temperature in the dark for 30 minutes. Proteins were digested overnight with 0.1 μg trypsin (Promega, Mannheim, Germany) in 25 mM ammonium bicarbonate at 37 °C as previously described [27]. Before MS analysis (see below), peptides from 3–4 gel pieces were pooled together resulting in three samples per gel lane. The peptides were dried in a speed-vac, solubilized in 0.1% (v/v) trifluoroacetic acid, desalted using C_18_ ZipTips (Merck Millipore, Darmstadt, Germany) according to the manufacturer’s instructions, dried again, and then analysed by liquid chromatography combined with mass spectrometry (LC-MS/MS) as described below.

### NanoLC-Orbitrap MS/MS analysis of tryptic peptides

Dried peptides were dissolved in 1% (v/v) formic acid, 2% (v/v) acetonitrile in water and injected into an Ultimate 3000 nano ultra high performance liquid chromatography system (Dionex, Sunnyvale CA, USA) connected to a Q-Exactive quadrupole-orbitrap mass spectrometer (Thermo Scientific, Bremen, Germany) equipped with a nano-electrospray ion source. Chromatographic separation was done using a nanoViper Acclaim PepMap 100 column (C_18_, 3μm, 100 Å) (Dionex, Sunnyvale CA, USA) with 50 cm bed length. The flow rate was 300 nL/min and the solvent gradient was 12% B to 43% B in 93 minutes, followed by further increase to 90% B in 6 minutes. Solvent A was 0.1% (v/v) formic acid in water and solvent B was 80% (v/v) acetonitrile, 0.08% (v/v) formic acid in water. The mass spectrometer was operated in data-dependent mode to switch automatically between orbitrap-MS and higher-energy collisional dissociation (HCD) orbitrap-MS/MS acquisition. The resolution was R = 70.000 and R = 35.000 for MS and MS/MS, respectively. For optimal acquisition of MS/MS spectra, automatic gain control (AGC) target values were set to 1.000.000 charges or a maximum injection time of 128 ms was allowed. Data dependent analysis was applied in order to isolate and fragment the 10 most intense ions at any given time throughout the chromatographic elution. These ion precursors were then excluded for further fragmentation for 20 seconds.

### Bioinformatics analysis

Raw files were imported into MaxQuant [26] version 1.4.1.2 for identification and label-free quantification (LFQ) of proteins. For protein identification in MaxQuant, the database search engine Andromeda [28] was used to search MS/MS spectra against the UniProt Complete Proteome Set of *E*. *faecalis* V583 (3,240 sequences) with a tolerance level of 6 ppm for MS and 20 ppm for MS/MS. Trypsin was used as enzyme and two missed cleavages were allowed. Carbamidomethylation of cysteines was set as a fixed modification and protein N-terminal acetylation, oxidation of methionines, deamidation of asparagines and glutamines and formation of pyro-glutamic acid at N-terminal glutamines were allowed as variable modifications. The ‘match between runs’ feature of MaxQuant, which enables identification transfer between samples based on accurate mass and retention time [25], was applied with a match time window of one minute and an alignment time window of 20 minutes. All identifications were filtered in order to achieve a protein false discovery rate (FDR) of 1%. Proteins were considered as “present” if they were detected by at least two peptides in at least two of the three biological replicates.

Protein quantification was based on the MaxQuant label-free algorithm [25], using both unique and razor peptides for protein quantification; at least 2 ratio counts were required for a protein quantification to be considered valid. Calculations of Benjamini-Hochberg corrections were done using the Perseus package (available at http://www.maxquant.org/). Protein abundance was calculated as a ratio of protein LFQ intensity and the sum of all LFQ intensities (total protein) in the sample, as previously described by Wisniewski and colleagues [29].

To predict secreted proteins we used a combination of three prediction algorithms. The SignalP server [30] version 4.1 (available at http://www.cbs.dtu.dk/services/SignalP/) was used with default settings for gram-positive bacteria to predict signal peptide cleavage sites. LipoP [31] version 1.0 (available at http://www.cbs.dtu.dk/services/LipoP/) was used to predict lipoproteins and TMHMM [32] version 2.0 (available at http://www.cbs.dtu.dk/services/TMHMM/) was used for prediction of transmembrane helices. Proteins predicted by TMHMM to harbour a single transmembrane helix located within the first 60 N-terminal amino acids where assumed to have a signal peptide. A protein was considered secreted if this was predicted by at least two of three prediction algorithms.

Information on the domain structure and potential function of uncharacterized proteins was extracted from Pfam version 27.0 (available at http://pfam.xfam.org/) and InterPro version 47.0 (available at http://www.ebi.ac.uk/interpro/). Operon structures were retrieved from ProOpDB [33] (available at http://operons.ibt.unam.mx/OperonPredictor/).

## Results and Discussion

**Fig 1:**
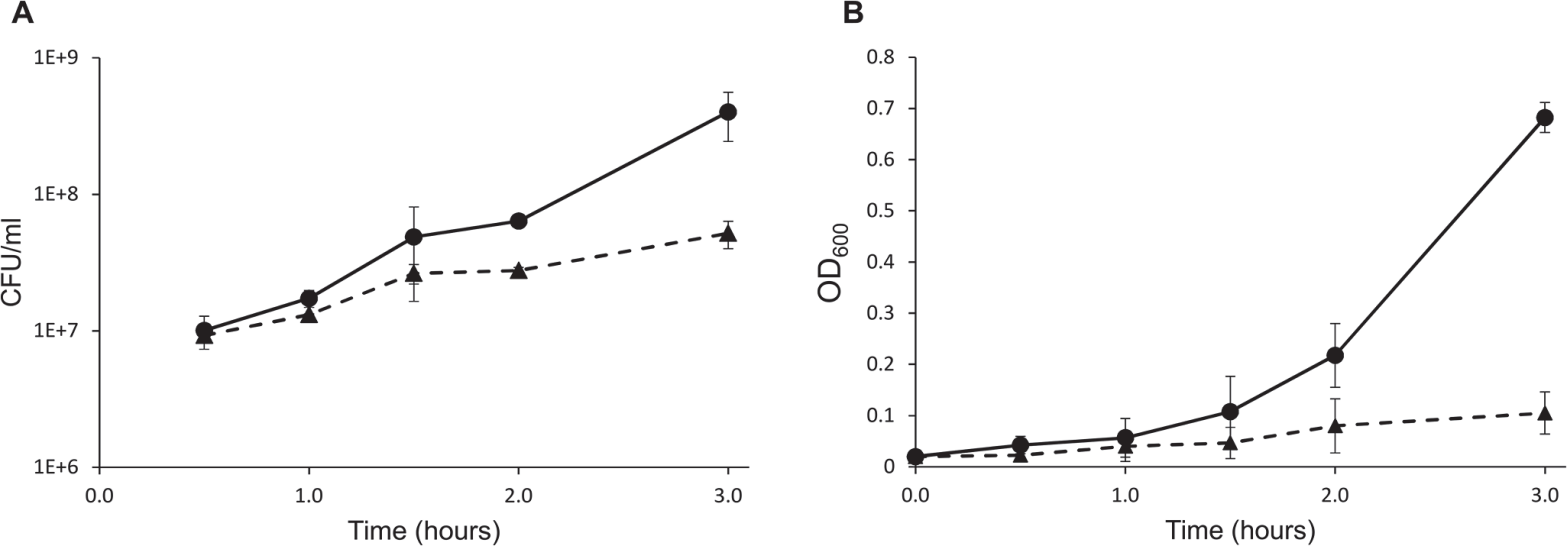
Growth curves. The curves show initial growth of *E*. *faecalis* after resuspending cells harvested from an overnight culture in 2xYT to OD_600_ ~0.02 in fresh medium, either 2xYT (circles on solid lines) or sterile urine (triangles on dashed lines). Growth was monitored by measuring colony forming units per milliliter (CFU/ml) (A) and optical density at 600 nm (OD_600_) (B). The data points correspond to the mean ± standard deviation of two individual experiments. Samples for proteomic analysis were collected after 1.5 hours.

*E*. *faecalis* V583 was pre-cultivated in the standard laboratory medium 2xYT and then diluted into either pre-warmed 2xYT or to pre-warmed urine. The cultures were further allowed to incubate for 1.5 hours before harvesting for proteomics analysis (Fig 1); at this point the growth rate started to differ between cultures with and without urine.

### Quantitative comparison of *E*. *faecalis* V583 secretomes

**Fig 2:**
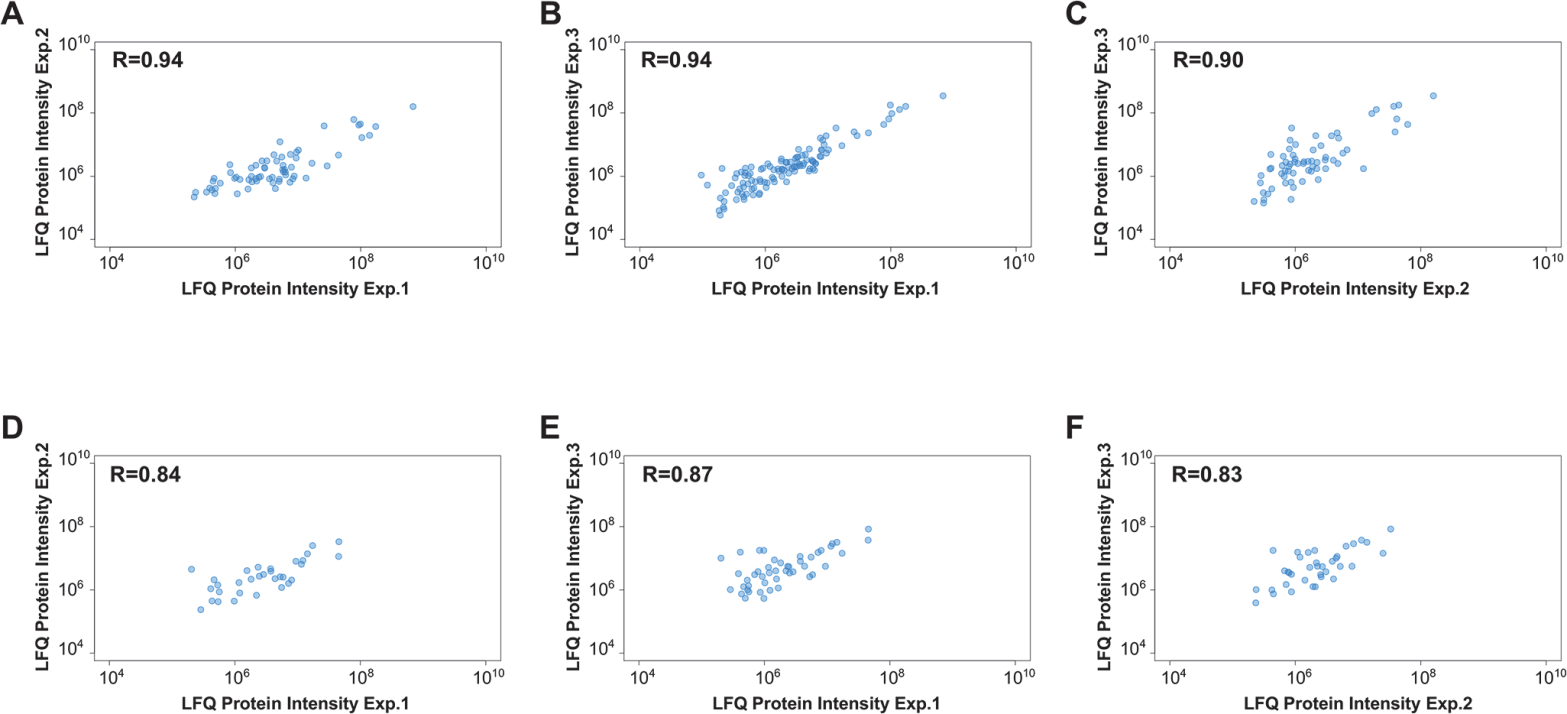
Quality of protein quantification. The figures show the pairwise reproducibility of label-free quantification (LFQ) between the replicates used for secretome quantification for the three 2xYT replicates (A-C) and the three urine replicates (D-F). Pearson correlations are indicated in each graph.

**Fig 3:**
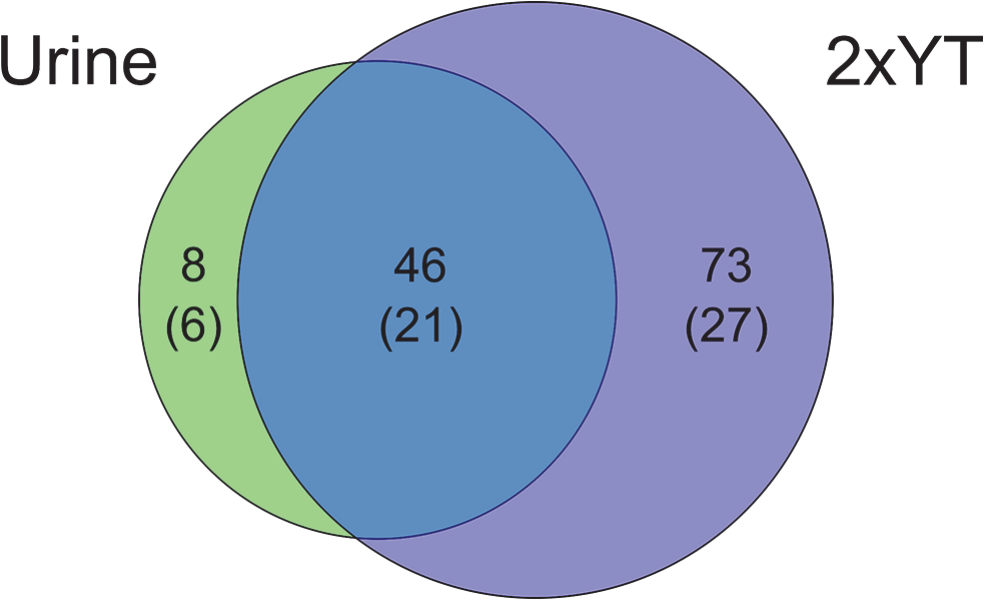
Proteins identified in the secretome of *E*. *faecalis* V583 when grown in 2xYT or urine for 1.5 hours. The Venn diagram shows the number of identified proteins in 2xYT, urine and in both conditions. The number of proteins predicted to be secreted is indicated in parenthesis.

To identify differentially expressed proteins, the secretomes of *E*. *faecalis* V583 incubated in control medium (2xYT) or urine were analysed using an MS-based proteomic approach. Precipitated proteins from cell free supernatants were separated by SDS-PAGE and in-gel digested using trypsin. The peptides were analysed by orbitrap LC-MS and identified/quantified using MaxQuant. The relative label-free quantification was highly reproducible between biological replicates, showing Pearson correlations between LFQ intensities varying from 0.83 to 0.94 (Fig 2A–2F), which are values similar to those observed in other LFQ studies [34]. Using a FDR of 1%, and after removal of contaminants, reversed hits and proteins identified with one peptide only, we identified 153 proteins represented by 2.235 peptide sequences. In line with common practice, another filter was applied, requiring a protein to be detected by at least two peptides in at least two of three biological replicates, in order for the detection to be considered significant. This led to a total of 127 proteins, which were distributed over the various samples as shown in Fig 3. The relatively low number of identified proteins reflects the fact that the bacterium was only allowed to grow for 1.5 hours (roughly to an OD_600_ of 0.1, Fig 1). The fact that fewer proteins were identified in the urine culture may partly be explained by human urine being a complex matrix that may bind proteins and that itself contains proteins in potentially high concentrations [35]. Urine proteins could mask identification of bacterial proteins in the data-dependent acquisition strategy used here, as only the most abundant ions are selected for fragmentation in the mass spectrometer. In addition, urine has been proposed to have limited amounts of iron, manganese and glucose [36] and under these conditions the bacterium may not utilize all pathways functional in a normal environment and thus produce fewer proteins.

The complete genome of V583 codes for 3240 putative proteins (Uniprot complete proteome set) of which 9% (294 proteins) are predicted as secreted when using the combination of SignalP, LipoP and TMHMM, as described in the Methods section. We detected 127 proteins, of which 43% (54 proteins) were predicted to be secreted. Proteomic analysis of the culture supernatant thus led to the expected enrichment of extracellular proteins, although datasets are inevitably polluted with intracellular proteins, as observed in other secretome studies [37, 38]. In total, 18% of all predicted secreted proteins in *E*. *faecalis* were detected, which is reasonable considering the results reported in other studies and the fact that one would not expect all predicted extracellular proteins to be secreted at 1.5 hours harvest time.

**Fig 4:**
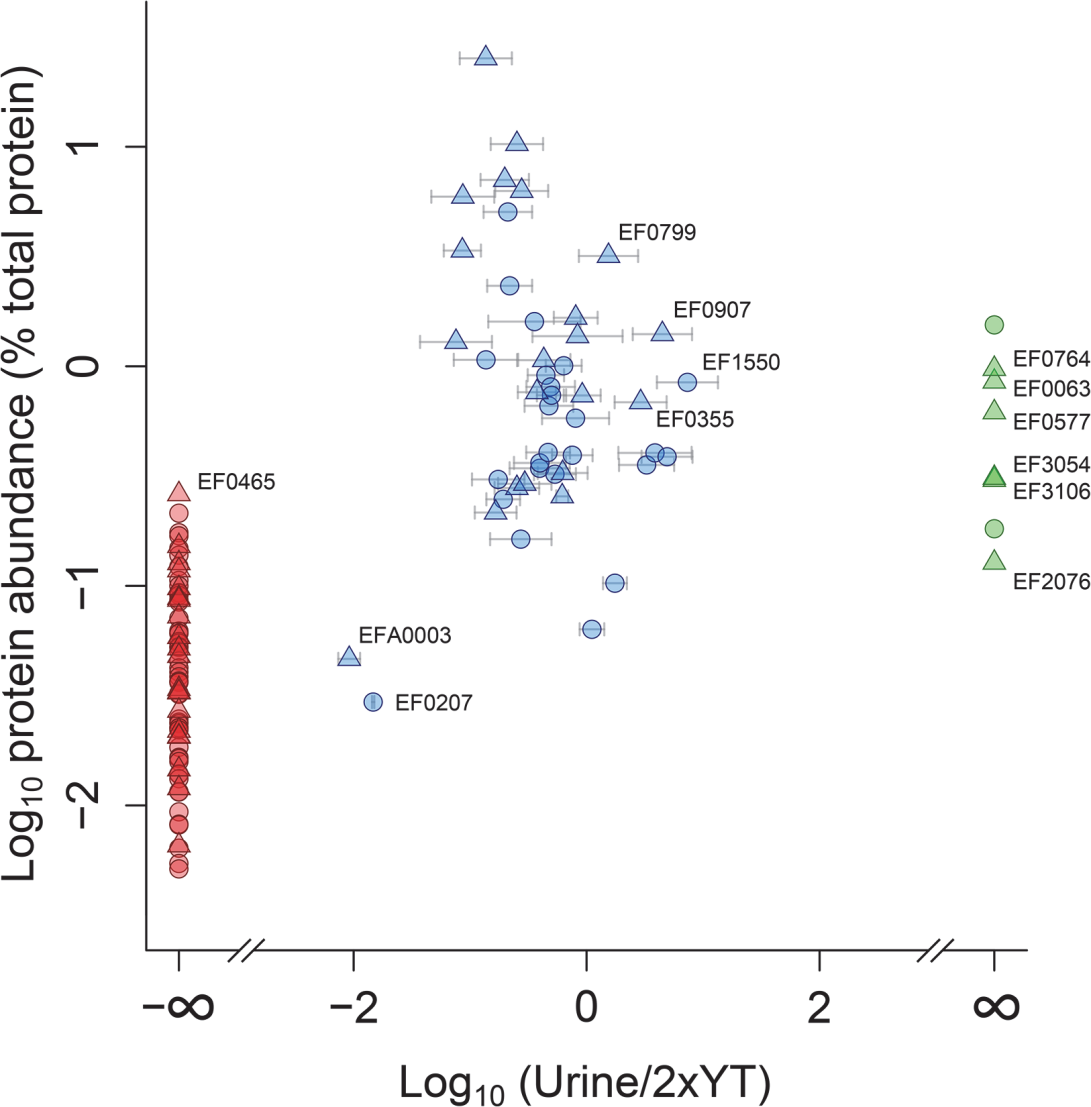
Quantitative analysis of proteins identified in the *E*. *faecalis* V583 secretome. The graph shows quantitative analyses of proteins identified exclusively in urine (green), in both urine and 2xYT (blue) and exclusively in 2xYT (red). For finite values, error bars displaying standard deviations of three replicates are shown. The X-axis shows the log ratio of the levels observed in urine and 2xYT (urine/2xYT) and the Y-axis shows protein abundance as a percentage of total protein. Proteins predicted to be secreted are shown as triangles while intracellular proteins are shown as circles. Proteins that are discussed in detail in the text are labeled. All eight proteins reported here as urine specific were identified and quantified with at least two peptides in at least two of three replicates. See S1 Table for details.

Looking only at the 54 detected proteins that are predicted to be secreted, 6 proteins were found exclusively in the urine culture (Table 1, Fig 4 green points, S1 Table), 27 proteins were exclusively found in the 2xYT culture (Fig 4 red points, S2 Table), and 21 proteins were found in both (Fig 4 blue points, S3 Table). Fig 4 shows that the most abundant proteins were present in both conditions while the least abundant proteins where predominantly those that were only detected in the 2xYT culture.

**Table 1:** Proteins with a predicted N-terminal signal peptide identified exclusively in the secretome of *E*. *faecalis* grown on urine.

UniProt Accession No.	Gene name	Protein name / Domain structure	Sequence coverage [%]	Protein abundance(% total protein)	Biological process (GO)
H7C720	EF0577	Adhesion lipoprotein	45.9	0.62	Metal ion transport; cell adhesion
Q832Z2	EF2076	Endocarditis specific antigen (EfaA)	9.7	0.13	Metal ion transport; cell adhesion
Q839T9	EF0063	Pheromone binding protein, putative	25.6	0.85	Transport
Q82ZF4	EF3106	Peptide ABC transporter peptide-binding protein	8.6	0.30	Transport
Q837R7	EF0764	Uncharacterized protein	43.1	0.97	
Q82ZK1	EF3054	Lipoprotein	15.4	0.31	

Domain structures were identified using Pfam and shown in S1 Table. EF0577 and EF2076 contains TroA domains, which are part of a periplasmic solute binding protein family; part of a family of ABC transporter metal-binding lipoproteins. EF0063 and EF3106 contains SBP_bac_5 domains, which are bacterial extracellular solute-binding proteins, believed to be involved in active transport of solutes across the cytoplasmic membrane. EF3054 contains two PepSY domains, which are thought to have a protease inhibitory function. For EF0764, Pfam does not find any known domains, nor does it annotate a signal peptide as with the other proteins, however both SignalP, LipoP and THMM indicate the presence of such a signal peptide. The functional annotation provided by Gene Ontology (GO) is based on inferences from the domain annotations (InterPro2GO). Additional information on these proteins is available in Table 2 and S1 Table.

Interestingly, while previous transcriptomics studies of enterococcal responses to urine have yielded lists of significantly up-/down-regulated proteins containing up to >700 candidates, the present rigorous proteomic analysis of the immediate response provides a much more limited list of potentially important proteins.

#### Proteins exclusively detected in the urine cultures

To be able to cause a clinical infection such as UTI, a bacterium needs to colonize the gut and periurethral mucosa and subsequently adhere to the uroepithelium. Indeed, of the six secreted proteins unique to cultivation in urine, two, EF0577 and EF2076, are homologous to known streptococcal adhesins such as SsaB from *Streptococcus sanguis* [39, 40] and FimA from *S*. *parasanguis* [41, 42]. EF0577 is currently annotated as “Adhesion lipoprotein” and is located in the pathogenicity island of V583. EF2076 is annotated as “Endocarditis specific antigen” (EfaA), a well-known virulence factor of *E*. *faecalis* originally detected in connection with studies of endocarditis [43, 44]. Using a mouse model system, it has been shown that an EfaA mutant of *E*. *faecalis* OG1RF is less virulent than the wild-type strain [44]. Both proteins, which share 60% sequence identity, contain a TroA domain (Pfam accession PF01297) belonging to a family of ABC transporter metal-binding lipoproteins. EF0577 and EF2076 are likely the metal binding surface lipoproteins of ABC transporters encoded by the *ef0575-ef0578* and *ef2074-ef2076* (*efaCBA*) operons. The transcriptional regulation of the two proteins is similar, both during incubation in urine (Table 2) and during growth in the presence of excess zinc or excess manganese [45].

**Table 2:** Previously observed regulation of proteins identified exclusively in the secretome of *E*. *faecalis* grown on urine.

UniProt Accession No. (Gene name)	Previously observed regulatory effects						
	Urine[1, 6]	Serum[1]	Bile[10]	SDS[10]	Bile+SDS[10]	Blood[11]	Erythromycin[9]
H7C720 (EF0577)	M: 10.6				V: 16.0		
	M: 17.1						
Q832Z2 (EF2076)	M: 4.9	M: 66 (qPCR)	V:3.7				V: -1.7
	M: 7.5	M: 5 (qPCR)					V: -2.1
	M: 89 (qPCR)						V: -2.8
	M: 2195 (qPCR)						
	O: 7.0						
	O: 10.6						
	S: 8.0						
	S: 9.2						
Q839T9 (EF0063)	M: 4.0					V: 5.7	
	M: 8.6						
	O: 26.0						
	O: 16.0						
	S: 5.7						
Q82ZF4 (EF3106)	M: 1.5					V: 68.6	V: -3.7
	M: 34.3						V: -2.1
	O: 1.7						
	O: 10.6						
Q837R7			V: -3.7	V: 6.1			
Q82ZK1	M: 1.3						

The table summarizes results from previous studies on enterococcal responses involving the proteins listed in Table 1. All data are derived from transcriptomics studies, except if stated otherwise. Only significant changes are included and the numbers are recalculated as fold change; positive numbers indicate up-regulation, negative numbers indicate down-regulation. In addition to the data shown here, regulation data exist for the effects of bivalent metal ions, showing up-regulation (Zn^2+^) or down-regulation (Cu^2+^, Mn^2+^) of EF0577 and EF2076 only [45], and NaCl, showing up-regulation of EF2076 [68]. Blank cells indicate lack of data. The strains used were V583 (V), MMH594 (M), OG1RF (O), and Symbiflor 1 (S). Additional q-PCR-based transcriptome studies, which are cited in the main text [12, 14], did not include quantitative data and are therefore not included in the Table. The results of these studies indicated that of the six proteins in the Table, EF2076 was up-regulated for the food strain LA160 during growth on urine. The other five of these six proteins were not tested/found in these studies.

While homologies with streptococcal adhesion proteins suggest roles of EF0577 and EF2076 in enterococcal adhesion, available experimental evidence for the functionality of these proteins is limited to their role in metal, in particular manganese, transport. Both the *efaCBA* operon and the *ef0575-0578* operon are regulated by the transcriptional regulator (EfaR), in a manganese-dependent manner [46, 47]. In a recent study by Abrantes et al., it was shown that EfaR is a major transcriptional regulator of manganese transporters in *E*. *faecalis* and that its mutations reduces the ability of *E*. *faecalis* to form biofilms, survive inside macrophages and tolerate oxidative stress, all properties associated with host colonization and infection [47]. The observed down-regulation of the *ef0577* and *ef2076* genes in response to excess manganese [45] further confirms their role in manganese transport, and could also explain the increased production of EF0577 and EF2076 in urine, where metal concentrations are low and high-affinity transporters thus are required for survival [48]. Interestingly, while the EfaR insertion mutant showed clearly weakened virulence traits, similar effects were not observed for insertion mutants of EF2076 (EfaA) or EF0577 [47], suggesting that the two proteins have overlapping functions. Combined with existing data, the present proteomic data clearly show that the EF0577 and EF2076 (EfaA) are of major importance for enterococcal interactions with its host, including in a urine environment. Further work to verify, characterize and analyse the functional implications of the predicted adhesin function of EF0577 and EF2076 is clearly needed.

In addition to metal ABC transporters, amino acid, peptide and amine ABC transporters are also believed to be linked to bacterial virulence [49]. We identified a lipoprotein (EF3106) annotated as a peptide-binding protein with an extracellular solute-binding domain (Pfam accession PF00496) covering 69% of the protein. EF3106 is part of an ABC transporter involved in peptide transport (the last in an operon of five genes) and the transcription of the *ef3106* is known to be up-regulated during growth in urine and blood (Table 2). Another lipoprotein found exclusively in the urine fraction is EF0063, harboring the same domain structure as EF3106, with the extracellular solute-binding domain covering 70% of the protein. However, although EF0063 possess similar domain structure as EF3106, its genetic context is very different. The *ef0063* gene is not located in an operon with other ABC transporter components. Interestingly CodY regulatory motifs are located upstream of *ef0063* in *E*. *faecalis* [50], meaning that expression of the gene might be regulated by CodY (EF1645 in V583), a protein known to be involved in adapting bacteria to stress and starvation and recognized as a global regulator of virulence-associated properties in several Gram-positive bacteria [51].

Lipoproteins in general are involved in many important cellular processes such as substrate-binding and-presenting to ABC transporters, folding of excreted proteins, pheromone production, and antibiotic resistance, as reviewed by Reffuveille et al. [49]. Another lipoprotein identified in the urine cultures only, EF3054, has no gene ontology annotation, but Pfam identifies two PepSY domains together covering 57% of the protein. PepSY domains comprise 60–90 amino acids and proteins harbouring PepSY domains typically contain a signal peptide or a transmembrane helix that would direct the proteins to the exterior of the cell wall [52]. Yeats *et al*. have shown that PepSY domains serve as inhibitors of members of the M4 family of metallopeptidases [52] and the ability of PepSY domains to regulate protease activity could be linked to pathogenicity [52]. The fact that we find this lipoprotein exclusively after incubation in urine suggests that regulation of protease activity plays a role in the adaptability or pathogenicity of V583. Transcriptomics studies also indicated up-regulation of this protein in response to a urine environment, although the effects were small (up-regulated by 1.3 fold, Table 2).

**Fig 5:**
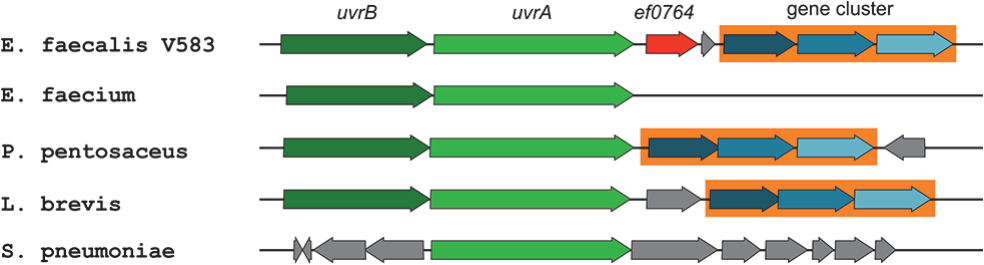
Gene context analysis. The figure shows the gene context around *ef0764* (indicated in red) as well as (potentially) related gene clusters in other gram-positive bacteria. Two genes upstream of *ef0764*, *uvrA* and *uvrB* (green), are involved in DNA repair and the downstream gene cluster of unknown function (orange box) commonly co-occurs with these known DNA repair genes. This cluster potentially encodes a predicted P-loop-containing kinase, a CofD-like protein and a cytoplasmic hypothetical protein. Genes in grey are genes that do not show similarity to other genes in this specific region of the genomes shown; while some have annotated functions, their genomic location is not conserved, nor associated with the location of *uvrA* and *uvrB*. The *ef0764* gene shows no similarity to the gene located in a similar position in *L*. *brevis*, and BLAST analysis could not identify any homologues in any other species.

The sixth secreted protein, EF0764, is a protein of unknown function, giving no hits in Pfam. The protein contains a signal peptide cleavage site and its identification in the supernatants of urine-incubated cells is evident, with six different unique peptides being detected giving 43% combined sequence coverage. Two of the same peptides were also found in one of the three replicates of the 2xYT culture (S1 Table), but in this case, peptide intensities were 14 times lower when compared to the peptides detected in samples from the urine culture. To our knowledge, regulation of this uncharacterized protein in response to a urine environment has not been reported before, but expression of its gene was found to be affected by bile (down, 3.7-fold) and SDS (up, 6.1-fold) (Table 2). Interestingly, EF0764 seems to be unique to *E*. *faecalis*, indicating that it could be exploited as a marker. Analysis of the genomic context and synteny using The SEED [53] (Fig 5) showed that *ef0764* is located upstream (separated by 72 nucleotides) of another small gene, *ef0765*, coding for a hypothetical protein of 55 amino acids belonging to the functionally uncharacterized YusW-like protein family, however, the two genes do not appear to be part of an operon structure. Two genes upstream on the same strand, *uvrA* and *uvrB*, are both important players in DNA repair [54]. Downstream on the same strand, there is a predicted gene cluster that commonly co-occurs with DNA repair genes. This cluster potentially contains a predicted P-loop-containing kinase (ATPase), a CofD-like protein that may be a 2-phospho-L-lactate transferase [48] and a cytoplasmic hypothetical protein belonging to COG1481, an uncharacterized protein domain. This conserved gene cluster has an operon structure, but its function is not known, nor why it co-occurs with DNA repair genes. DNA damage can result from oxidative stress, and both proteins (UvrA and UvrB) involved in DNA repair mechanisms and antioxidant defense systems are known virulence factors in *E*. *faecalis* [55, 56]. Perhaps, the hitherto uncharacterized EF0764 protein is involved in DNA repair or protection from oxidative stress imposed by the host. More targeted experiments on this protein need to be conducted to elucidate its potential function in *E*. *faecalis* adaptation and pathogenicity.

### Proteins exclusively detected in the 2xYT cultures

Most proteins identified exclusively in the 2xYT medium (Fig 4 red points; S2 Table) were present in low amounts and not obviously of interest in relation to pathogenicity and survival. The most abundant secreted protein only found in the 2xYT culture is EF0465, which is a transcriptional regulator belonging to the LytR family. In accordance with this observation, transcriptomics studies by Vebø et al. [6] showed that transcription of *ef0465* was down-regulated 1.7–1.9 fold during growth in urine, for all three *E*. *faecalis* strains tested, MMH594, OG1RF and Symbiflor.

### Proteins detected in both urine and the 2xYT cultures

Proteins occurring in the supernatants of both cultures are quantitatively visualized in Fig 4 (see full list in S3 Table) and include secreted and intracellular proteins present in intermediate to high amounts. The majority of these proteins are hydrolases and many have cell wall/membrane associated functions such as peptidoglycan degradation (S3 Table).

Significance B calculations in Perseus using a Benjamini-Hochberg correction at 5% reveals two proteins standing out as significantly down-regulated in urine. One of these is secreted TraC (EFA0003; *traC-1*), with a suggested role in pheromone-binding [57], while the other is a ribosomal protein with a predicted intracellular location (EF0207; *rplD*). The significance of these two identifications is questionable, because imputed intensities had to be used for calculating ratios, due to the low abundance in the urine samples (see S3 Table for details).

The protein with the highest degree of up-regulation on urine was EF1550, a histone-like DNA binding protein, which shares 82% homology with the streptococcal histone-like protein HlpA. Interestingly, in streptococci, HlpA, which is predicted to be cytoplasmic, has been detected on the cell surface, where it forms complexes with lipoteichoid acid (LTA) and mediates bacterial adherence by also interacting with heparan sulfate proteoglycans of host cells [58, 59]. Binding of HlpA-LTA complexes to host cells is known to stimulate production of pro-inflammatory cytokines in cultured murine macrophages, suggesting a role of HlpA in promoting tissue damage at infection sites [60]. To our knowledge, regulation of EF1550 in response to a urine environment has not been reported before.

Three secreted proteins were up-regulated in urine, EF0799 (*atlA*; Autolysin), EF0355 (*atlB*; Peptidoglycan hydrolase / Endolysin) and EF0907 (Peptide ABC transporter peptide-binding protein). EF0907 harbours an extracellular solute-binding domain (Pfam accession PF00496) similar to EF0063 and EF3106 (discussed above, see Table 1) and is involved in transport of peptides. The primary function of EF0799 is thought to be peptidoglycan degradation, in particular during digestion of the septum after cell division, and this protein has been linked to enterococcal biofilm formation [61] and antibiotic sensitivity [62]. EF0355 has a similar activity and mutation studies have shown that it can act as a surrogate for EF0799 [63]. A third hydrolytic enzyme related to EF0799 and EF0355, EF1992 (*atlC*), was not detected in this study.

### Concluding remarks

In this proteomics study, we have compared the secretome of *E*. *faecalis* during the initial phase of cultivation in urine with the secretome during cultivation in standard laboratory medium, 2xYT. This led to the identification of extracellular proteins and a histone-like “moonlighting” protein, which are all putatively involved in early phases of infection processes in the urinary tract. It must be emphasized that urine is a poor nutrient source [1] and that during development of an UTI, the bacteria are likely to feed on glycoproteins of the UT epithelium. This source of additional energy was not present in our experiments. Thus, while our study reveals interesting proteins likely to be directly or indirectly involved in UTI development, these proteins do not tell the full story.

Known *E*. *faecalis* virulence factors include gelatinase (GelE) and serine protease (SprE), two proteins regulated via the Fsr two-component signal transduction system that mediates quorum sensing-based responses [64]. Shankar et al. [65] have shown that the secretomes of Fsr-positive and Fsr-negative strains of *E*. *faecalis* grown on BHI medium show distinct differences, beyond the mere presence of GelE and SprE in the Fsr-positive strains. In our study, using an Fsr-positive strain [66], GelE or SprE were detected neither in the urine nor in the 2xYT secretome, indicating that quorum activation had not occurred in any of the two conditions. We detected 17 of the 34 unique proteins detected by Shankar et al. in the Fsr-negative strain (one on urine only, the cytoplasmic EF1167, 7 on 2xYT only and 9 in both). In contrast, we detected only three of the 25 unique proteins detected by Shankar et al. in the Fsr-positive strain (two on 2xYT only, and one on both). Thus, the conditions used in this study may seem to mimic an Fsr-null mutant.

The proteins detected include a known virulence factor, EfaA, and a homologue, EF0577. These two proteins, as well as two of the other detected urine-specific proteins are involved in solute transport, which conceivably is important for survival in the urine environment. Adhesion seems to be important too, since EfaA and EF0577 have putative roles in this respect, whereas streptococcal HlpA, a homologue of up-regulated EF1550, is a known adhesion factor. The detection of EF0764 is of interest because this protein is highly specific for *E*. *faecalis*, has a completely unknown function, and had previously not been associated with the urine response, whereas current data show a clear association. The fact that the transcript encoding this protein was not detected in a comprehensive transcriptome analysis of *E*. *faecalis* cultivated in urine is remarkable and may indicate unusual regulation justifying further investigations. The rigorous proteomics approach applied here illustrates the power of label-free quantification in combination with bacterial culturing in human urine as a method for finding candidates potentially responsible for UTI by *E*. *faecalis*.

## Supporting Information

S1 Table Proteins identified in the secretome only after cultivation in urine. (XLSX)

S2 Table Proteins identified in the secretome only after cultivation in 2xYT. (XLSX)

S3 Table Proteins identified in the secretome in both conditions. (XLSX)
